# The findings on the CEUS of diffuse large B cell lymphoma in abdomen: A case report and literature review

**DOI:** 10.3389/fonc.2023.1093196

**Published:** 2023-02-02

**Authors:** Yu-Qing Zhang, Xin-Yue Wang, Ying Huang

**Affiliations:** Department of Ultrasound, Shengjing Hospital of China Medical University, Shenyang, China

**Keywords:** non-Hodgkin’s lymphoma, ultrasound, contrast-enhanced ultrasound (CEUS), time-intensity curve (TIC), Positron Emission Tomography- Computed Tomography (PET- CT)

## Abstract

**Background:**

PET-CT is the first choice for the imaging diagnosis of intraperitoneal lymphomas. Contrast-enhanced ultrasound (CEUS) is rare in the diagnosis of intraperitoneal nodal lymphoma.

**Case summary:**

A 62-year-old man was admitted for examination with “right upper abdominal pain”. Ultrasound was used to refer to the masses in the hilar region, spleen, and anterior sacral region respectively. The masses were all hypoechoic, and blood flow signals could be detected by CDFI. Laboratory tests of CA125 were within normal limits. CEUS examination was performed on the three masses respectively. The three masses showed different perfusion patterns. Thickened vessels appeared around the mass in the hilar region, a peripheral centrally directed perfusion pattern was observed in the splenic mass, and blood supply vessels appeared in the center of the presacral mass with a significant filling defect. They all showed a contrast pattern with rapid clearance and hypoenhancement compared with the surrounding areas. Ultrasound guided needle biopsy revealed non-Hodgkin’s lymphoma, diffuse large B-cell lymphoma, non-germinal center origin. After biopsy, the patient was treated with R-CHOP regimen for chemotherapy, and the tumor disappeared by routine ultrasound review after 5 cycles of chemotherapy.

**Conclusion:**

To the best of our knowledge, this report is the first to describe the findings of CEUS in intraperitoneal nodal lymphoma. CEUS has various manifestations in intraperitoneal nodal lymphoma. Future studies are still needed to explore the diagnostic features of CEUS in intraperitoneal nodal lymphoma.

## Introduction

The incidence of lymphoma continues to rise worldwide. According to Global Cancer Statistics 2018, non-Hodgkin’s lymphoma ranks 13th and 11th among all forms of malignancy in terms of morbidity and mortality (1). The diagnosis of lymphoma depends on pathology, and imaging examination can provide more staging information. The application of contrast-enhanced ultrasound (CEUS) in the diagnosis of lymphoma is rare in medicine. Intraperitoneal lymph node lymphoma is less common than superficial lymph nodes. Up to the present, no studies have reported the diagnosis of intraperitoneal nodal lymphoma by contrast-enhanced ultrasound.

## Case presentation

### Chief complaints

A 62-year-old male patient was admitted to the hospital because of “Pain in the right upper abdomen”. Through CT upper abdominal plain scan, a mass in the hilar region could be seen clearly. Thus, further examination was recommended.

### History of past illness

The patient had no medical history of other blood diseases, no low fever, no night sweats, and no significant weight loss. In 2015, he underwent cerebrovascular stenting for basilar artery stenosis, and recovered well after surgery. Rosuvastatin, Clopidogrel and Aspirin were taken orally to control the disease. In 2016, he underwent cholecystectomy due to gallstones, and no abdominal discomfort occurred after the operation. He had a history of hypertension for more than 20 years, and his blood pressure was well controlled without other chronic diseases at ordinary times.

### Personal and family history

The patient smoked for more than 20 years and quit smoking for more than 10 years. Denied any history of alcohol use. No history of drug or food allergy.

### Physical examination

There was a palpable mass in the anterior sacral area, which was hard and slightly tender. No tenderness, rebound pain, muscle tension in the rest of the place. All other vital signs were stable, blood pressure was not high, and no positive signs were detected.

### Laboratory examinations

The patient’s serum β2 microglobulin was 2.58mg/L at admission, which was higher than the normal value (0.7-1.8mg/L). Urinary β2 microglobulin 0.762 mg/L, higher than the normal value (&lt; 0.24mg/L) thymidine kinase (TK1)0.33pmol/L, still in the normal range. Serum CA125 was 10.58U/mL, and there was no obvious abnormality.

### Imaging examinations

**Figure 1:**
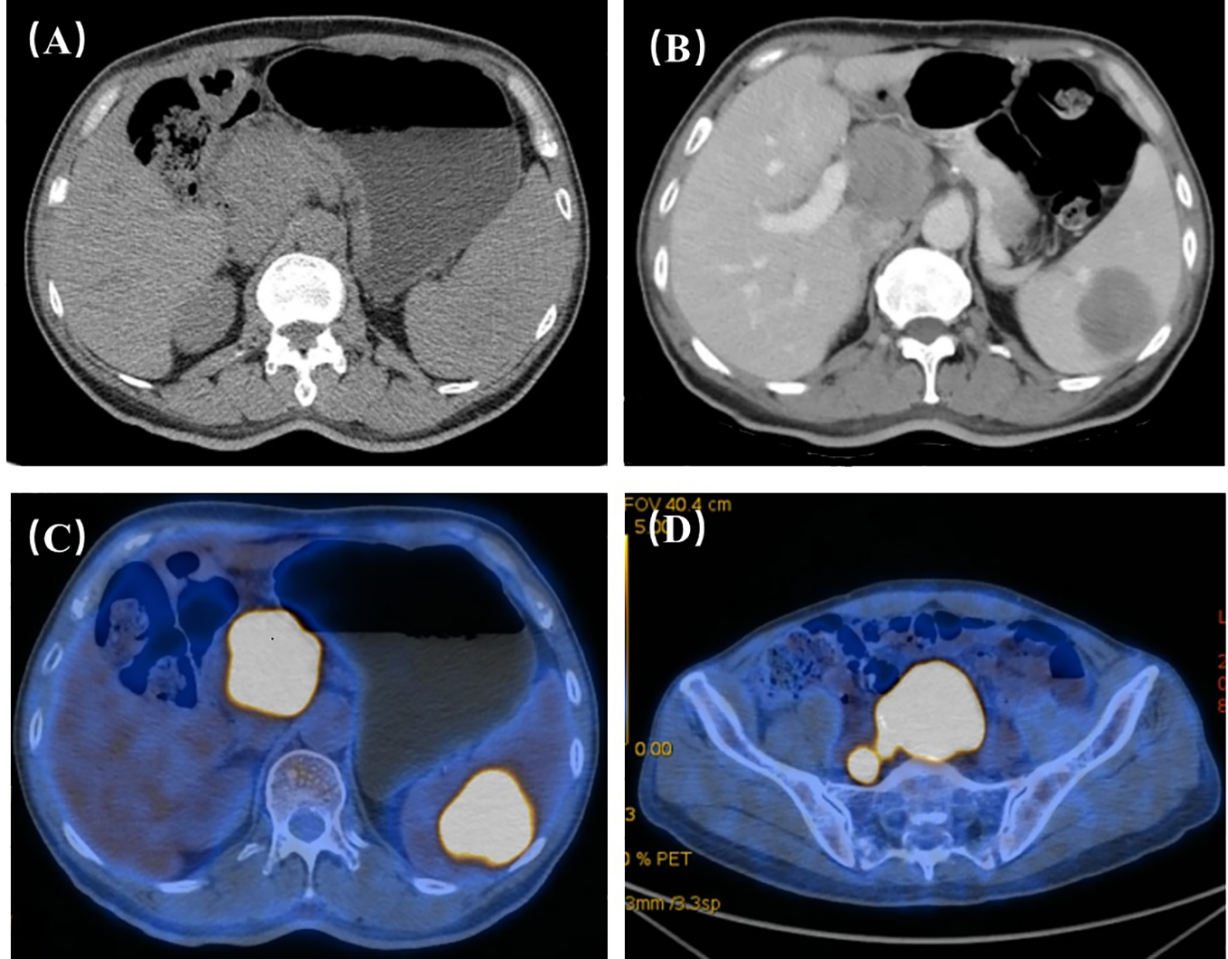
**(A)** Through plain scan of the upper abdomen, it clearly showed the masses in the hilar region, but the masses in the spleen were not obvious. **(B)** Through contrast-enhanced CT, it could show the masses in the hilar region and splenic region, and the masses showed progressive and uneven enhancement. The hilar region nodules: plain scan 30HU arterial phase, 40HU portal vein phase, 52HU delayed phase, 65HU; spleen nodules: plain scan 35HU arterial phase, 46HU portal vein phase, 52HU delayed phase, 63HU; upper abdominal enhancement suggested: Further examination is recommended for hilar and splenic masses. **(C, D)** FDG metabolism of hilar mass, splenic mass and presacral mass on PET-CT increased, and finally PET-CT suggested that the three were homologous and had a high possibility of lymphoma.

**Figure 2:**
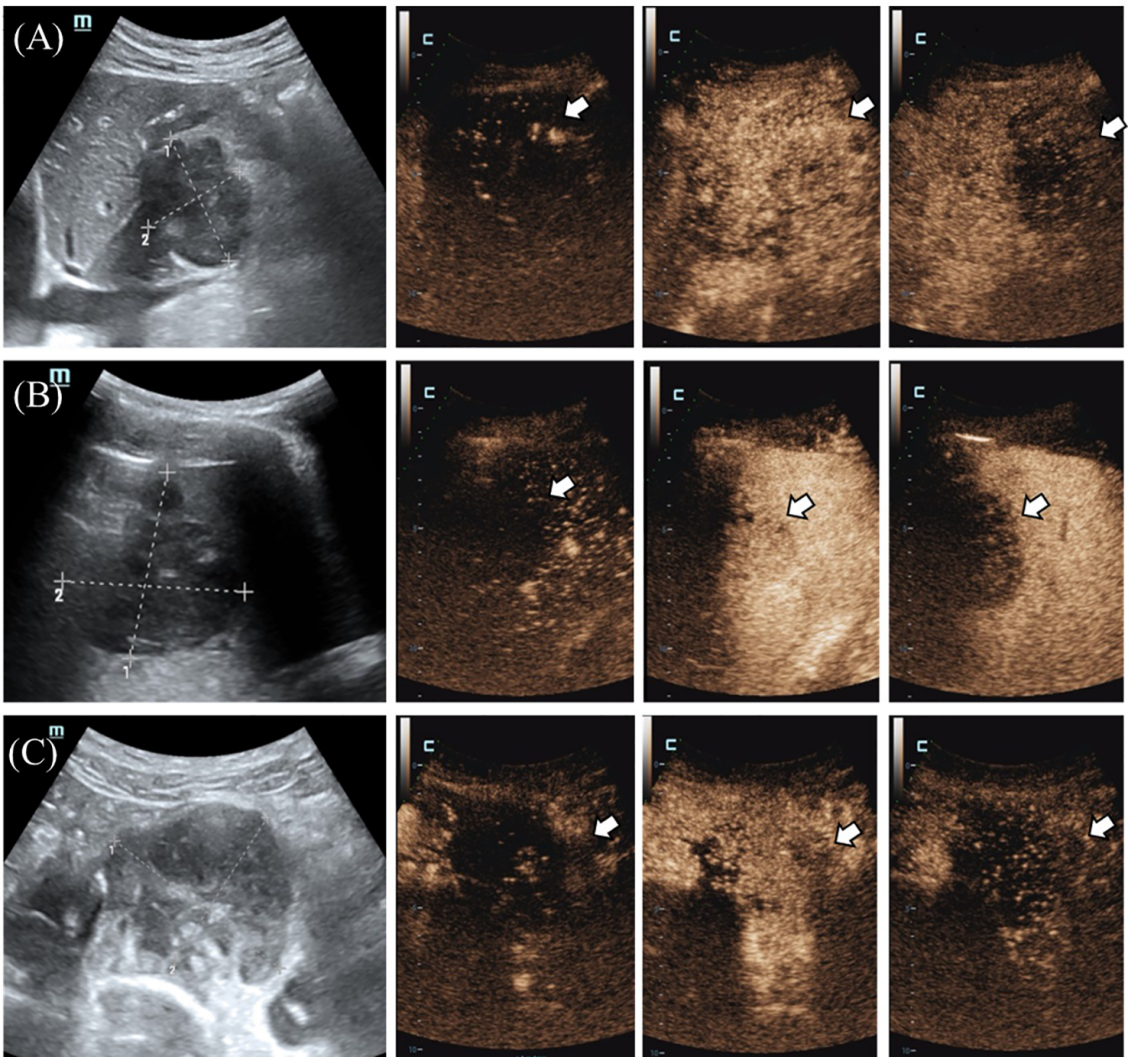
**(A)** The tumor in the hilar region showed thickened blood vessels, located at the edge of the tumor (indicated by the arrow), and was enhanced and cleared earlier than the surrounding tissue. **(B)** The splenic mass (shown by the arrow) showed a peripheral to central enhancement pattern with uneven hypoenhancement, which was enhanced later and cleared earlier than normal splenic tissue. **(C)** In the presacral mass (shown by the arrow), the central vessels of the mass were enhanced first, and the enhancement began later than the peripheral tissues, with uneven hypoenhancement, and there were obvious filling defects, and the clearance was earlier than the peripheral tissues.

Because a hilar mass was showed after scanning the upper abdomen through CT, the patient was scheduled for further examination through contrast-enhanced ultrasound (**Figure 1**). The ultrasound examination on abdomen was performed using a Resona9 ultrasound system (Mindray Medical International, China) equipped with an SC6-1U (1-6 MHZ) transducer. Conventional ultrasound examination showed a hypoechoic mass in the hilar region, the size of the mass was 4.65x5.33x3.91cm, the boundary was clear, the shape was irregular, and CDFI could detect blood flow signals. Another nearly-circular hypoechoic mass was found in the spleen. The size of the mass was 4.96x4.74x5.19cm, the boundary was clear, and the blood flow signal could be detected by CDFI. Another hypoechoic mass was seen in the anterior sacral area, with a size of 5.84x5.20x4.68cm, located below the bifurcation of the abdominal aorta, with clear boundary and irregular shape. There was no adhesion with the surrounding intestine. Blood flow signals could be detected by CDFI. Further CEUS diagnosis was recommended by the patient’s physician and informed consent was obtained. Depth, gain, and focus are thoroughly adjusted to achieve optimal visualization according to the radiologist’s habits. The timer was activated after a high-dose injection of 1.5 mL of Sonovue (Bracco, Italy) suspension (an ultrasound contrast agent) and 5 mL of saline (Bracco, Italy). CEUS examination was performed on all three masses. After conducting contrast-enhanced ultrasound, it showed that the blood vessels were thickened at the edge of the tumor in the hilar region, which were enhanced earlier than the surrounding tissues. The tumor showed diffusing snowflake enhancement inside, and showed uneven hypoenhancement after reaching the peak (compared with the liver tissue), and was rapid wash-out compared to that of the liver tissue. The splenic mass showed a peripheral enhancement pattern to the center, with uneven hypoenhancement. The enhancement was later than that of the normal spleen tissue, and rapid wash-out compared to normal spleen. There was no significant change in the lesion range after CEUS compared with the two-dimensional ultrasound. In the presacral mass, the central vessels of the mass were enhanced first, and the enhancement began later than the peripheral tissues, with uneven hypoenhancement. And there were obvious filling defects, and the clearance was earlier than that of the peripheral tissues. The boundary between the three masses and surrounding tissues was obvious (**Figure 2**). The time-intensity curve (TIC) showed that the ascending slope of the lesion was higher than that of the surrounding tissue, although the lesion was delayed hypoperfusion, which also suggested that the lesion area had more blood vessels and less resistance. The clearance time of the lesion was significantly earlier than that of the surrounding tissue, which was consistent with the characteristics of malignancy. The three masses did not have the same pattern of enhancement, and there were obvious filling defect areas in the presacral mass, while the enhancement was more uniform in the hilar region and the splenic mass. Although the enhancement patterns of the three masses were different, they all showed rapid wash-out, which was consistent with the characteristics of malignant masses. Among them, the thickened vessels at the edge of the tumor in the hilar region were consistent with the CEUS features of lymphoma in previous studies (2). The CEUS pattern of splenic mass was basically consistent with the characteristics of nodular splenic infiltration of malignant lymphoma mentioned in the literature. The CEUS pattern of central vessel enhancement first in the presacral mass has not been confirmed in the literature in lymphoma, but considering that the patient had no medical history of other malignant tumors and considering homology with other masses. Finally, the patient was diagnosed lymphoma under the CEUS pattern combined with the patient’s medical history.

### Pathological findings and immunohistochemical staining

Puncture biopsy was performed on the patient’s anterior sacral mass, and 16G automatic puncture biopsy needle (Bard, America) was used. The final pathological results suggested non-Hodgkin B-cell lymphoma, diffuse large B-cell lymphoma, and non-germinal center origin. Immunohistochemical results showed that CD20, CD21, CD19 were positive, Ki-67>90%, Bcl-2>90%, Bcl-6>80%.

### Results of flow cytometry

Through flow cytometry, the results showed double clonal B mature lymphocytes.

## Final diagnosis

The final diagnosis of this case was intranodal type diffuse large B-cell lymphoma with splenic infiltration.

## Treatment

The patient was diagnosed with diffuse large B-cell lymphoma and received R-CHOP chemotherapy, namely rituximab, dexamethasone, vincristine, epirubicin, and cyclophosphamide, for a course of 8 cycles. After 4 cycles of chemotherapy, abdominal enhanced CT examination showed that the size of the hilar lesion was about 2.96x2.11cm, the presacral mass was unclear, and the size of the splenic mass was about 2.79x3.04cm. After 5 cycles of chemotherapy, conventional ultrasound scan of the whole abdomen showed no definite lesions.

## Discussion

Diffuse large B-cell lymphoma (DLBCL) is the most prevalent subtype of non-Hodgkin lymphoma. Patients usually presented with progressive lymphadenopathy, extranodal disease, or presented with the both, which require treatments (3). The definitive diagnosis of lymphoma clinically depends on the detailed examination of tumor tissue. In addition to morphological characteristics, the accurate classification of lymphoma also requires specialized tests, including immunohistochemistry, flow cytometry, fluorescence *in situ* hybridization (FISH) and molecular testing (4, 5). Positron Emission Tomography-Computed Tomography (PET-CT) was used to evaluate organ involvement and clinical staging (6). However, PET-CT is often limited by the high cost of examination and the high radiation dose. In recent years, many studies have focused on more accurate diagnosis of lymphoma by imaging methods, further differentiating from other space-occupying lesions and prognosis evaluation (7–9). Different treatments from the most malignant tumors together with the negative prognosis, the timely diagnosis of lymphoma becomes necessary.

CEUS has the advantages of convenience and easy operation. Contrast-enhanced ultrasound can display the changes of microcirculation perfusion in real time. At present, contrast-enhanced ultrasound has been widely used (10). CEUS is rarely reported in the diagnosis of lymphoma compared with other diseases. Many studies in the literature focus on the CEUS features and differential diagnostic efficacy of superficial lymph nodes (11–13). It is rare to study abdominal lymph nodes.

In this case, under the CEUS mode the distribution of microvessels presented by different masses was different. Thickened blood vessels appeared around the mass in the hepatic portal area, which became the first perfusion area of the tumor. It may be caused by surrounding blood vessels in the invasive growth process of the tumor. The volume of the pre-sacral mass was large, and the rapid growth rate led to the lack of blood supply in the tumor. Due to the above the filling defect inside the mass was found by contrast-enhanced ultrasound. Due to the different growth location and size, the microvascular distribution of each homologous tumor is slightly different.

Superficial lymph nodes are the most common site of lymphomas, especially those in the head and neck. Contrast-enhanced ultrasound can clearly show the distribution of blood vessels in lymph nodes, which makes the diagnosis of superficial lymph nodes more accurate (**Table 1**). The studies on CEUS diagnosis of superficial lymph nodes are more mature. The study of Shan-Shan Yin et al. suggested that the CEUS findings of lymphoma are significantly different from those of lymph node metastasis and reactive lymph nodes. Through CEUS findings of lymphoma, it can mostly be seen the diffusing and even enhancement, and rarely be seen the non-perfusion filling defect areas. Lymph node metastases usually presented with centripetal enhancement, and non-perfusion filling defect area is more common. Reactive hyperplastic lymph nodes presented with uniform hyperenhancement due to vascular hyperplasia, and lymph node tuberculosis presented with unclear lymph node boundary (11). Much of the literature comes to similar conclusions (14–16). This may be related to angiogenesis and vascular distribution in the lesion. Due to the rapid growth rate of malignant metastases, immature neovascularization and non-vascular necrotic areas are common in metastatic lymph nodes, which hinders the distribution of contrast agent to these areas then leads to perfusion defects. In most lymphomas, blood vessels are highly hyperplastic, which makes the microvesicles of contrast agent easy to flow and rapidly distribute throughout the lesion, resulting in more uniform enhancement (16). Although the features of contrast-enhanced ultrasound in lymphoma have been unanimously agreed in many literatures, there are still some exceptions. In the study, Ming Yu et al. reported 6 cases of lymphoma without perfusion (17). Due to the uncertainty in the qualitative manifestations of contrast-enhanced ultrasound in lymphoma, literature studies have made focus on the quantitative analysis of contrast-enhanced ultrasound. Shan-shan Yin et al. also analyzed the arrival time parameters of contrast media and found that compared with lymphoma, the contrast had arrived earlier in metastatic cancer but spent longer to the center (11). Xiaoyan Niu et al. suggested that quantitative indicators of CEUS were correlated with PET-CT indicators, which had potential diagnostic value for lymphoma (18). CEUS has a high diagnostic value in the diagnosis of benign and malignant superficial lymph nodes, which has been confirmed by studies. However, CEUS still manifested similarities in lymph nodes with different pathologies, and the treatment of lymph node diseases is completely different. Therefore, more studies are devoted to further explore the more accurate diagnosis of lymphoma by CEUS. Liu SR et al. injected subcapsular contrast injection and observed that lymph nodes of lymphoma patients had lymphatic vessel distortion and uneven distribution of contrast agent, which was completely different from reactive proliferative lymph nodes with uniform distribution of contrast agent and lymph node metastasis with local concentration of contrast agent and lymphatic vessel rupture (19). Compared with intravenous contrast medium injection, subcapsular contrast medium injection has a better effect in identifying the types of lymph nodes, it requires higher technical requirements for the operator. Thus, this kind of injection cannot be popularized wider. The prognosis of different subtypes of lymphoma varies greatly. According to the growth pattern and prognosis of lymphoma, it can be divided into aggressive lymphoma and indolent lymphoma. Studies suggest that the contrast-enhanced ultrasonography of indolent lymphoma is more similar to that of reactive proliferative lymph nodes, presenting rapid uniform hyperenhancement (13). The later the stage, the more aggressive and malignant the lymphoma, the richer the blood vessels in the tumor, the higher the blood flow velocity, and the higher the RI. The difference of ΔT and AS between early and advanced lymphomas was statistically significant, suggesting that advanced lymphomas had abundant blood flow at the CEUS quantitative level (20). However, there was no significant difference in contrast-enhanced ultrasound performance between HL and NHL. Further analysis of the various subtypes of lymphoma by contrast-enhanced ultrasound is not available. Studies suggest that contrast-enhanced ultrasound has a high application value in evaluating the response of lymphoma to drugs after chemotherapy, and it can accurately respond to changes in the blood supply of the lesion after chemotherapy. For the lesions that responded to chemotherapy, the area under the curve of CEUS quantitative analysis before and after chemotherapy showed statistically significant difference in perfusion index (PI) (21). in the study of M. Kumagawa et al., peak enhancement (PE) and PI are considered to be the effective indicators for evaluating whether a patient has achieved a complete response after chemotherapy (22). The prediction of lesion response after chemotherapy can quickly make clear the next treatment plan of patients more quickly.

**Table 1:** Contrast-enhanced ultrasound characteristics of superficial lymph node lymphoma reported in the literature.

Ref.	Year	Country	Type of literature	Gender(M/F)	Age	lymphoid neoplasms	CEUS feature	Conclusion	Contrast agent/dosage
Shan-shan Yin et al.	2018	China	Research	71/74	53.1 ± 14.4	Not specified	The peripheral arrival time of lymphoma is significantly later than that of metastatic cancer lymph nodes, the central arrival time is earlier than that of metastatic cancer lymph nodes, and the ΔT is significantly shorter than that of metastatic cancer lymph nodes.	CEUS can provide additional information for the differential diagnosis of superficial lymph node enlargement.	SonoVue/1.0ml
Leopoldo Rubaltelli et al.	2004	Italy	Research	28/17	27-76	Not specified	CEUS of lymphoma shows intense but heterogeneous speckle-like enhancement in the early arterial phase.	CEUS is highly accurate in the differential diagnosis of benign and malignant lymph nodes.	Esatune/4.8ml
Ji Nie et al.	2020	China	Research	76/67	53.4 ± 12.9	Not specified	Quantitative analysis of CEUS parameters showed that the PI (and AUC were lower in lymphomas than in cancerous lymph nodes. Lymphomas tend to show homogeneous enhancement	Contrast-enhanced ultrasound is used for the differential diagnosis of head and neck lymphomas and cancerous lymph nodes.	SonoVue/2.4ml
Ming Yu et al.	2009	China	Research	48/46	46	Not specified	Of the 17 lymphomas, 9 showed homogeneous hyperenhancement and 6 showed absence of perfusion.	Contrast enhancement patterns and temporal intensity profiles provide valuable diagnostic information for the differential diagnosis of benign and malignant lymph nodes.	SonoVue/2.4ml
Xiaoyan Niu et al.	2018	China	Research	42/21	50	Not specified	In the current lymph nodes, ΔI, AUC, and AS showed positive associations with PET-CT values.	The real-time enhancement process and TIC parameters can provide comprehensive information regarding the activity of lymph nodes for further examination.	SonoVue/2.4ml
Wenbin Jiang et al.	2018	China	Research	42/21	50	Not specified	When lymphoma CEUS shows ΔT < 5.5 or AS > 2.58, it indicates advanced stage and aggressiveness of the lymphoma	The quantitative parameters of CEUS help to assess the different clinical and pathological patterns of extramedullary lymphoma.	SonoVue/2.4ml
Xuelei Ma et al.	2018	China	Research	43/18	50.8	Not specified	83.1% of lymphomas present with diffuse uniform hyperenhancement	The rapid distribution of highly enhanced patterns in CEUS can be a useful diagnostic criterion for both aggressive and non-aggressive lymphomas.	SonoVue/2.4ml
Shirong Liu et al.	2019	China	Research	18/14	40.67 ± 16.83	Not specified	Subperitoneal CEUS showed that the majority (6 of 7, 85.7%) of lymphomas exhibited heterogeneous perfusion and lymphatic tract distortion.	subcapsular CEUS may help to distinguish between benign and malignant lesions	SonoVue/2.0ml
Lei Xin et al.	2017	China	Research	12/23	50.5	Not specified	Quantitative CEUS parameters before and after the first three cycles of chemotherapy showed significant differences in AreaΔ, PIΔ, and IΔ in the overall response group compared to the no responsive group.	The results of this study suggest that quantitative analysis of CEUS may be a useful method for assessing treatment response to superficial lymph node enlargement in lymphoma before and after chemotherapy	SonoVue/2.4ml
M. Kumagawa et al.	2021	Japan	Research	18/9	66	Not specified	Accordingly, changes in the ratio of PE < 1.09 and changes in the ratio of PI < 1.65, distinguished CR from non-CR accurately.	Changes in perfusion parameters assessed by CEUS may be able to assess the prognosis of patients with lymphoma	Daiichi-Sankyo/0.5ml

CEUS is relatively rare in the diagnosis of lymphomas in extranodal organs (**Table 2**). Through CEUS, it can well reflect the blood supply of the lesion and the distribution of microvessels, and has a good diagnostic efficiency for the differentiation of lymphoma from benign nodules of spleen (23–25). Lymphoma of the spleen is more common in secondary lymphoma invasion and formation, but primary lymphoma is rare to be seen. The findings of primary splenic lymphoma are similar to those of secondary CEUS, with early isoenhancement and early regression (26, 27). Other malignant tumors of the spleen are rare to be seen, and the differentiation of splenic lymphoma from other malignant tumors has not been reported. Christian Gorg et al. believe that CEUS has no added value in the diagnosis of splenic involvement in lymphoma and cannot improve the diagnostic accuracy of conventional ultrasound for splenic involvement (28). CEUS has been used to identify space-occupying lesions of the liver. Intrahepatic lymphoma, as an uncommon intrahepatic malignant tumor, is less common than other malignant tumors. Similar to the spleen, liver lymphomas secondary to intra nodal lymphomas is usually to be seen, primary liver lymphomas are rare. Primary liver lymphoma is often associated with HBV and HCV virus infections. HBV infection-related MALT was more common in primary liver lymphoma (29). More studies are needed to determine whether viral infection affects the progression of liver lymphoma. Color Doppler ultrasound often shows multiple vascular channels in malignant liver lymphoma, which is called “vascular penetration sign”. Contrast-enhanced blood vessels were first observed in the arterial phase of CEUS, and there were still angiograms in the Kupffer phase. This feature is significantly different from the typical contrast-enhanced ultrasound findings of other liver malignant tumors. However, liver malignant lymphoma also has rich blood supply CEUS findings that are not similar to other malignant tumors and cannot be differentiated from other diseases (30, 31). C. Trenker et al. also concluded that the differential diagnosis of hepatic malignant lymphoma from other malignant tumors could not be completed by CEUS in their study of CEUS findings in 38 cases of lymphoma (32). The renal lymphoma was mainly nodular type. It presented mostly hypoenhancement or isoenhancement in the arterial phase and hypoenhancement in the parenchymal phase through CEUS. Contrast-enhanced ultrasound has certain value in the differential diagnosis of renal lymphoma and benign nodules (33). The findings of CEUS in lymphoma with extranodal organ invasion are relatively uniform, and there is little difference between different extranodal organs. There are also extranodal organs with specificity through CEUS, and the literature studies are mainly based on case reports. One case serially reported the contrast-enhanced ultrasound findings of 6 cases of intrapulmonary lymphoma. 83% of the lymphomas were mainly supplied by pulmonary artery, which was not consistent with the findings of other pulmonary malignancies (34). There is a correlation between thyroid lymphoma and nodular Hashimoto’s thyroiditis. Lulu Yang et al. found that the combination of CEUS features and quantitative indicators has a good diagnostic efficacy in differentiating thyroid lymphoma from nodular Hashimoto’s thyroiditis (35), However, more studies are needed to confirm whether it can be widely used in clinical practice. Primary testicular lymphoma is rare, and it is difficult to distinguish lymphoma from other testicular lesions by conventional two-dimensional ultrasound or color Doppler. Literature studies have suggested that testicular lymphoma is mostly characterized by rapid high-enhancement contrast-enhanced ultrasound, which is more extensive than gray-scale ultrasound. An 80% increase in blood flow shows a straight vessel pattern with nonbranched increased vascular hyperplasia (36, 37). Most of the studies related to the diagnosis of extranodal organ lymphoma by CEUS are limited to the differentiation of other space-occupying lesions from benign or malignant. However, it seems that it is still difficult to distinguish lymphoma from other malignant tumors by CEUS. Contrast-enhanced ultrasound does not appear to contribute to the diagnosis of extranodal lymphoma subtypes.

**Table 2:** Contrast-enhanced ultrasound characteristics of lymphoma involving extranodal organs reported in the literature.

Ref.	Year	Country	Type of literature	Gender(M/F)	Age	Organ involved	lymphoid neoplasms	CEUS feature	Conclusion	Contrast agent/dosage
Marco Picardi et al.	2009	Italy	Research	53/47	30/32	spleen	Hodgkin lymphoma	After contrast material injection, most malignant nodules had clear intralesional vessels with isoenhanced and fine intralesional microcirculation with hypoenhanced appearance in the parenchymal phase.	Harmonic compound US with contrast enhancement for the characterization of possible nodules provides a higher sensitivity in the detection of splenic involvement by Hodgkin lymphoma.	Sonovue/2.4ml
Stefano Ballestri et al.	2014	Japan	Case	F	73	spleen	Not mentioned	Arterial phase at 15 s. initially, inhomogeneous parenchymal enhancement. Splenic lesions are isoechoic compared to the surrounding parenchyma. Parenchymal phase at 35 s. Hypoenhancement of the lesion.	CEUS was able to correctly identify as malignant.	Sonovue/Not mentioned
Marco Picardi et al.	2022	Italy	Research	43/34	48	spleen	Not specified	The enhancement pattern of lymphoma characterized by isoenhancement in the arterial phase followed by wash-out appearance that had early onset and marked degree.	It is important indications of CEUS for assessing the spleen at risk of lymphomatous invasion.	Sonovue/2.4ml
Tom Sutherland et al.	2010	Australia	Case	M	66	spleen	diffuse large B cell lymphoma	In the early phase, the mass demonstrated homogenous enhancement similar to the surrounding normal spleen and was isoenhanced for the first 20 seconds. Rapid wash-out then occurred with the mass being hypoenhanced relative to normal spleen in the parenchymal phase and becoming subsequently.	With second-generation sonographic contrast agents, the enhancement characteristics of the primary splenic lymphoma in our case were identical to those of secondary splenic lymphoma described in the literature with early contrast enhancement and rapid progressive washout.	Definity/1.5ml
Zhang W et al.	2022	China	Research	20/29	31-73	spleen	Not specified	It mostly showed uniform enhancement when checking Splenic lymphoma by CEUS, which could be low or high enhancement, but not septal enhancement within lesions. Spacer enhancement can be used to distinguish splenic lymphoma from splenic tuberculosis.	Contrast-enhanced ultrasound is valuable for the differential diagnosis of splenic lymphoma and splenic tuberculosis.	SonoVue/2.4ml
Christian Görg et al.	2009	Germany	Research	27/24	20-83	spleen	Not specified	During the arterial phase focal lesions were hypoenhancement or isoenhancement, During the parenchymal phase focal lesions were hypoenhancement.	CEUS has no clear advantage for diagnosis of splenic lymphoma involvement.	Sonovue/2.4ml
Francesco Giuseppe Foschi et al.	2010	America	Case	M	62/58	liver	MALT	Contrast-enhanced ultrasonography showed that the nodular lesion had mild inhomogeneous hyperenhancement in the arterial phase and wash-out in the portal and late phases.	There is no imaging pattern on CEUS is specific for PHL.	Sonovue/2.4ml
Yuki Yamashita et al.	2021	Japan	Case	F	66	liver	MALT	After Sonazoid injection, the tumor was depicted as a homogeneously hyper-enhanced lesion with penetrating vessels in the early arterial phase, which began to wash out in 15 seconds. The tumor was hypo-enhanced in the portal and late phases on CEUS.	Primary hepatic extranodal marginal zone lymphoma of MALT presenting image findings different from those of typical hepatocellular carcinoma along with the vessel penetration sign.	Sonazoid/Not mentioned
C. Trenker et al.	2013	Germany	Research	22/16	60.1	liver	Not specified	The present study shows hypoenhancement in the portal phase in n = 36 (94.7%) cases. In n = 2 (5.3%) cases hepatic lymphoma presented isoechogenic enhancement in the portal phase. In n = 38 (100%) cases lymphoma lesions exhibited hypoenhancement during the late phase.	The application of CEUS can help to find the right diagnosis. A discrimination between malignant liver lesions, such as liver lymphomas, metastasis or HCC, remains impossible.	Sonovue/2.5ml
Corinna Trenker et al.	2015	Germany	Research	M	75	Pulmonary	Not specified	pulmonary lymphomas surprisingly showed a PA vascularization pattern in all but 1 case. the typical”wash-out” sign that has been understood to be a sign of malignancy was detected in 3 patients, whereas 3 patients also showed isoechoic parenchymal enhancement.	Definite differentiation from other malignant or benign pulmonary lesions cannot be achieved by CEUS	Sonovue/2.4ml
Lulu Yang et al.	2021	China	Research	8/23	65	Thyroid	Not specified	For most of the PTL lesions, contrast agent entered in a centripetal way and presented as hypoenhancement, as well as heterogeneous. In CUES mode, PTL had significant lower values of PI and AUC than those of NHT.	CEUS is an efficient diagnostic tool in the differential diagnosis of PTL and NHT for patients with diffuse HT.	Sonovue/2.0ml
Li Yang et al.	2022	China	Case	M	59	Testicular	Not specified	the PTL lesions presented by a hyperenhancement by enlarged range in CEUS along with a nonbranching linear vascular pattern on microvascular US.	Combined with straight vascular signs on grey-scale US and painless testicular mass of physical examination, it can provide some help for early non-invasive diagnosis of PTL.	Sonovue/2.4ml
Guntram Lock et al.	2016	Germany	Case series	M	59	Testicular	Not specified	On CEUS, all lymphoma lesions show marked hyperenhancement.	the key sonographic features of testicular lymphoma are as follows:(1) sharply demarcated homogeneous hypoechoic testicular lesions with marked hypervascularization on colorcoded sonography; (2) a rapid (<7 seconds) filling time of contrast bubbles; and (3) a straight parallel course of intralesional vessels in most cases, as shown by contrast-enhanced sonography.	Sonovue/2.4ml

The value of CEUS in the diagnosis and prognostic assessment of superficial intra nodal lymphoma has been validated. However, its diagnostic value for lymphoma with extranodal organ invasion seems to need further research and verification.

## Conclusion

CEUS findings of this case of intraperitoneal nodular lymphoma showed hypoperfusion in the early stage of enhancement compared with the delayed perfusion of surrounding tissues, and rapid clearance. The pattern of lesion initiation enhancement is varied. To the best of our knowledge, this report is the first to describe CEUS findings of intraperitoneal nodal lymphoma. CEUS is rarely used in the diagnosis of lymphoma compared with other diseases. The value of CEUS for intraperitoneal nodal lymphoma still needs to be confirmed by subsequent studies.

## Data availability statement

The original contributions presented in the study are included in the article/Supplementary material. Further inquiries can be directed to the corresponding author.

## Ethics statement

Written informed consent was obtained from the individual(s) for the publication of any potentially identifiable images or data included in this article.

## Author contributions

YZ: Paper writing, patient information collection, literature review; XW: Paper writing, information collection; YH: Writing instruction, implementation of intervention, information collection. All authors contributed to the article and approved the submitted version.
